# The Differential Expression of Long Noncoding RNAs in Type 2 Diabetes Mellitus and Latent Autoimmune Diabetes in Adults

**DOI:** 10.1155/2020/9235329

**Published:** 2020-02-19

**Authors:** Zhang Pengyu, Yan Yan, Fu Xiying, Yang Maoguang, Li Mo, Cheng Yan, Shen Hong, Wang Lijuan, Zhang Xiujuan, Cai Hanqing

**Affiliations:** Department of Endocrinology, The Second Hospital of Jilin University, Changchun, Jilin Province, China

## Abstract

**Background:**

Long noncoding RNAs (lncRNAs) were previously found to be closely related to the pathogenesis of diabetes.

**Objectives:**

To reveal the differentially expressed lncRNAs and messenger RNAs (mRNAs) involved in type 2 diabetes mellitus (T2DM) and latent autoimmune diabetes in adults (LADA) and predict the lncRNA target genes to derive their expression profiles for the diagnosis of T2DM and LADA and their differential diagnosis.

**Methods:**

Twelve venous blood samples were collected from T2DM patients, LADA patients, and nondiseased subjects to obtain total RNAs. After removing rRNA from total RNAs to establish the desired library for sequencing, quality control and quantification analyses were carried out. The fragments per kilobase of exon model per million reads mapped (FPKM) of lncRNAs were calculated to construct the gene expression profiles of lncRNAs and mRNAs. Fold changes (fold change: 2.0) and *p* values (*p* values (

**Results:**

Compared to nondiseased controls, 68,763 versus 28,523 lncRNAs and 133 versus 1035 mRNAs were significantly upregulated and significantly downregulated, respectively, in T2DM patients. For LADA patients, 68,748 versus 28,538 lncRNAs and 219 versus 805 mRNAs were significantly upregulated and significantly downregulated, respectively, relative to nondiseased controls. Compared to T2DM patients, 74,207 versus 23,079 lncRNAs and 349 versus 137 mRNAs were significantly upregulated and significantly downregulated, respectively, in LADA patients. Based on the correlation analysis, seven lncRNA-mRNA pairs (BTG2, A2M, HECTD4, MBTPS1, DBH, FLVCR1, and NCBP2) were significantly coexpressed, and two lncRNAs (ENST00000608916 and ENST00000436373) were newly discovered.

**Conclusion:**

Significant differences in lncRNA expression were discovered among the three groups. Furthermore, after predicting lncRNA expression profiles, GO/KEGG pathway analysis could deduce the target gene function.

## 1. Introduction

Currently, adult onset, slow progression, and evidence of damage to one or more islet autoantibodies serve as the three major characteristics of latent autoimmune diabetes in adults (LADA). The autoimmune process of LADA is slower than that of the classical type 1 diabetes mellitus (T1DM) and new therapeutic interventions might cause a delay in *ß* cell failure. Early diagnosis is thus crucial for the treatment of LADA [1]. Long noncoding RNAs (lncRNAs) are a type of ncRNAs with transcripts exceeding 200 nucleotides in length [2]. LncRNAs can be classified as sense lncRNAs, antisense lncRNAs, bidirectional lncRNAs, intronic lncRNAs, and intergenic lncRNAs, according to their functions and biological sites [3]. LncRNAs, with exosomes as vectors, are targeted for transportation to recipient cells and function as endogenous lncRNAs that participate in intercellular signaling and specifically alter the expression of proteins and genes in recipient cells [4]. LncRNAs have been revealed to mainly function in the following four processes: chromatin, transcriptional regulation, posttranscriptional regulation, and translation level [5]. As lncRNAs are closely related to the pathogenesis of diabetes [6], we aimed to reveal the differential expression of lncRNAs and mRNAs in type 2 diabetes mellitus (T2DM) and LADA and predict the target genes of lncRNAs to derive their expression profiles for the diagnosis of T2DM and LADA and their differential diagnosis.

## 2. Materials and Methods

### 2.1. Ethical Affirmation

This trial followed the ethical principles of the Ministry of Health Ethical Review of Biomedical Research Involving Human Beings (2016), WMA Helsinki Declaration (2013), and CIOMS International Ethics Guide for Human Biomedical Research (2002) and Good Clinical Practice (GCP), guided by the Code of Quality Management for Clinical Drug Trials, in accordance with the requirements of a program approved by the Ethics Committee in order to ensure the scientific nature of the study and protect the health and rights of the subjects.

### 2.2. Grouping

By using the protocols and consent procedures approved by the Ethics Committee of Second Hospital of Jilin University, 12 individuals that visited this hospital between July 2017 and June 2018 were recruited to participate in the current study. These individuals were patients with T2DM (*n* = 4), LADA patients (*n* = 4), and nondiseased individuals (*n* = 4). In this retrospective study, samples were collected in advance. Thereafter, we applied for an exemption from informed consent and obtained approval from the Ethics Committee. The diagnostic criteria included patients diagnosed with diabetes according to the World Health Organization criteria. The selection criteria for the LADA group were patients diagnosed with diabetes between 30 and 55 years of age who were insulin-independent for at least 6 months after diagnosis and positive for glutamate decarboxylase autoantibodies (GAD Ab) or tyrosine phosphatase autoantibodies (IA2 Ab). Patients diagnosed with diabetes at 30–55 years of age and negative for autoantibodies in diabetes were enrolled in the T2DM group. Normal controls were enrolled in the nondiseased group. The cases excluded were 6 cases of endocrine diseases other than DM, 5 cases of untreated hypertension, 4 cases of autoimmune diseases, 3 cases of other acute or chronic inflammatory diseases, 2 cases of liver and kidney dysfunction, and 1 case of malignant tumor.

### 2.3. Sample Preparation, Total RNA Extraction, and RNA Quality Control

Blood samples from LADA and T2DM patients and nondiseased controls were collected and centrifuged for 10 min at 3000 rpm and 4°C to separate and collect serum. Samples were subsequently refrigerated at −80°C prior to analysis. After total RNAs were obtained, rRNAs were removed using the Ribo-Zero rRNA Removal Kit (MRZG12324; Illumina, USA). Thereafter, the RNA was pretreated with the TruSeq Stranded Total RNA Library Prep Kit (batch no. 20020596; Illumina, USA) to establish the desired library for sequencing. Quality control and quantification of the library were performed using the Bioanalyzer 2100 instrument (SK-07017; Agilent Technologies, USA).

### 2.4. Bioinformatics Analysis

To construct the gene expression profiles of lncRNAs and mRNAs, we calculated their fold changes and the *p* value and FPKM of the lncRNAs at the transcript level and mRNAs at the gene level for three sets of samples [7]; the Cuffdiff software (v2.2.1) was used. We screened for the differentially expressed lncRNAs and mRNAs (fold change: 2.0, *p* value: 0.05, FPKM ≥ 0.1). Furthermore, to obtain the significantly coexpressed lncRNA-mRNA pairs, the target genes of lncRNAs were deduced according to the association between adjacent sites. The mRNAs in these coexpressed lncRNA-mRNA pairs were considered to be targets of the corresponding lncRNAs. When a coexpressed pair was not detected, the mRNA closest to the lncRNA was considered to be its target. The functions of the target genes of lncRNAs were subsequently predicted by GO/KEGG pathway analysis.

### 2.5. Quantitative Real-Time Polymerase Chain Reaction (qRT-PCR)

The expression of lncRNAs was confirmed by quantitative real-time polymerase chain reaction (qRT-PCR). Three upregulated and five downregulated lncRNAs were selected for verification. The housekeeper gene, GAPDH, was used as a reference for normalization. All primer sequences are listed in Table 1. Total RNA was reverse-transcribed into cDNA using the PrimeScript RT Kit (#RR037 A; Takara, Osaka, Japan) according to the manufacturer's instructions. The obtained cDNA was analyzed by qRT-PCR and connected with the EVA green qPCR mixture (MasterMix-ER; ABM, Vancouver, BC, Canada) in a biological RADCFX real-time PCR system. Three independent samples were tested. The relative expression rate of lncRNA was measured by the 2^−ΔΔCT^ method.

### 2.6. GO and KEGG Pathway Analyses for the Selected lncRNAs

The differentially expressed lncRNAs-related genes were analyzed by gene ontology (GO) and Kyoto Encyclopedia of Genes and Genomes (KEGG) pathway analyses. GO analysis consists of three parts, namely, molecular function, biological process, and cell component. Conversely, KEGG pathway is a reference base for mapping molecular data sets in genomics, transcriptome, proteomics, and metabonomics to the KEGG pathway diagrams to explain the biological functions of these molecules. The KEGG pathway of the differentially expressed lncRNA adjacent target genes was analyzed to annotate and speculate the pathways involving these lncRNAs. A *p* value < 0.05 was considered to indicate statistical significance.

## 3. Results

A total of 97,286 lncRNAs and 20,308 mRNAs were detected in the three groups, with 12,463 lncRNAs and 15,162 mRNAs in the nondiseased group, 10,878 lncRNAs and 13,797 mRNAs in the T2DM group, and 9,013 lncRNAs and 14,537 mRNAs in the LADA group. When compared, seven significantly coexpressed pairs (BTG2, A2M, HECTD4, MBTPS1, DBH, FLVCR1, and NCBP2) and two newly discovered lncRNAs (ENST00000608916 and ENST00000436373) were identified.

### 3.1. Differentially Expressed lncRNAs and mRNAs and GO/KEGG Pathway Analysis of lncRNAs That Target mRNAs

**Figure 1:**
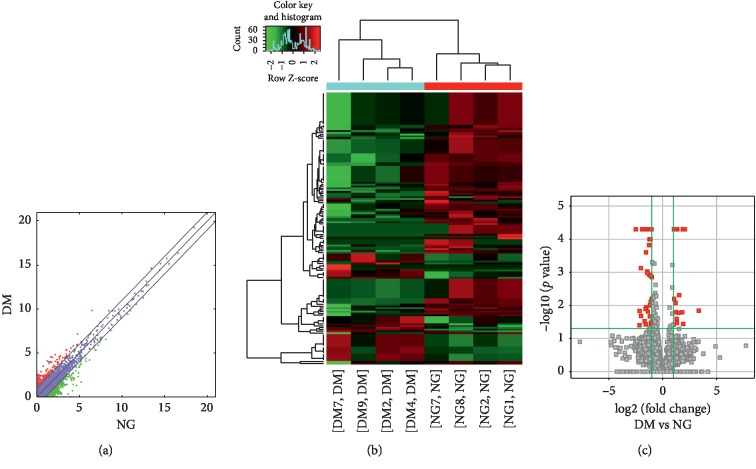
Changes in the expression profiles of lncRNAs in the T2DM and nondiseased groups. (a) The scatter plot reveals that a significant difference existed in the distribution of lncRNAs between the T2DM group and the nondiseased group. (b) The heat map depicts the hierarchical clustering of altered lncRNAs in the T2DM group compared to the nondiseased group. Red represents upregulation, while green represents downregulation. (c) The volcanic map displays the upregulated and downregulated lncRNAs in the T2DM group compared to the nondiseased group. T2DM—type 2 diabetes mellitus; lncRNAs—long noncoding RNAs.

**Figure 2:**
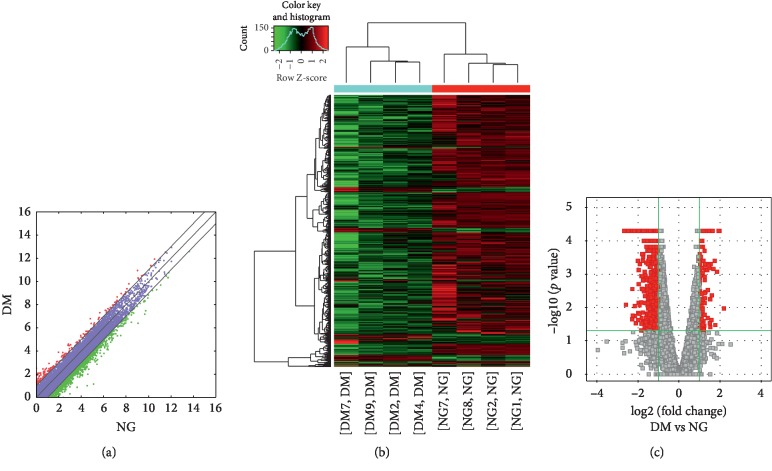
Changes in the mRNA expression profiles of the T2DM and nondiseased group. (a) The scatter plot reveals that a significant difference existed in the distribution of mRNAs between the T2DM group and the nondiseased group. (b) The heat map depicts the hierarchical clustering of altered mRNAs in the T2DM group compared to the nondiseased group. Red represents upregulation, while green represents downregulation. (c) The volcanic map displays the upregulated and downregulated mRNAs in the T2DM group compared to the nondiseased group. T2DM—type 2 diabetes mellitus; mRNAs—messenger RNAs.

**Figure 3:**
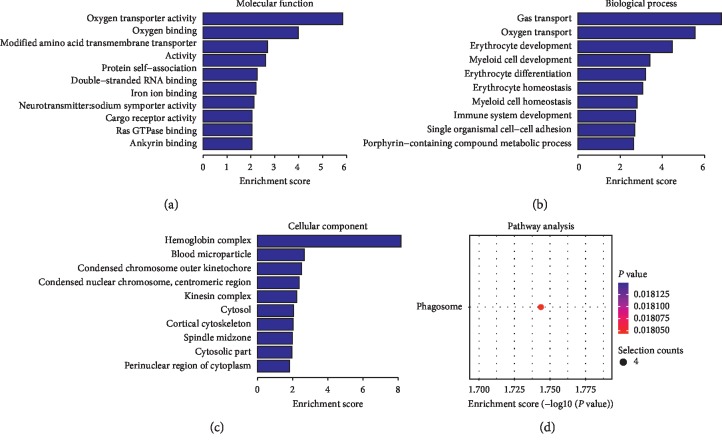
GO and KEGG signaling pathways for the lncRNAs that are differentially upregulated and target mRNAs. (a) Molecular function: oxygen transporter activity; oxygen binding function. (b) Biological process: red blood cell development and gas transport. (c) Cellular component: hemoglobin complex. (d) Analysis of the KEGG signaling pathways of lncRNAs that target mRNAs: phagosome signaling pathways. LncRNAs—long noncoding RNAs; mRNAs—messenger RNAs; GO—gene ontology; KEGG—Kyoto Encyclopedia of Genes and Genomes.

Compared to the nondiseased group, 68,763 lncRNAs were significantly upregulated and 28,523 lncRNAs were significantly downregulated (Figures 1(a)–1(c)), while 133 mRNAs were significantly upregulated and 1035 mRNAs were significantly downregulated (Figures 2(a)–2(c)) in the T2DM group. The expression changes for three lncRNAs (ENST00000364558, ENST00000608916, and ENST00000565382) were detected by qRT-PCR. Of the upregulated lncRNAs, the fold change of ENST00000364558 was the most significant. Based on GO and KEGG signaling pathway analyses, the lncRNAs that target mRNAs are mainly involved in the phagocytic signaling pathway (Figure 3).

**Figure 4:**
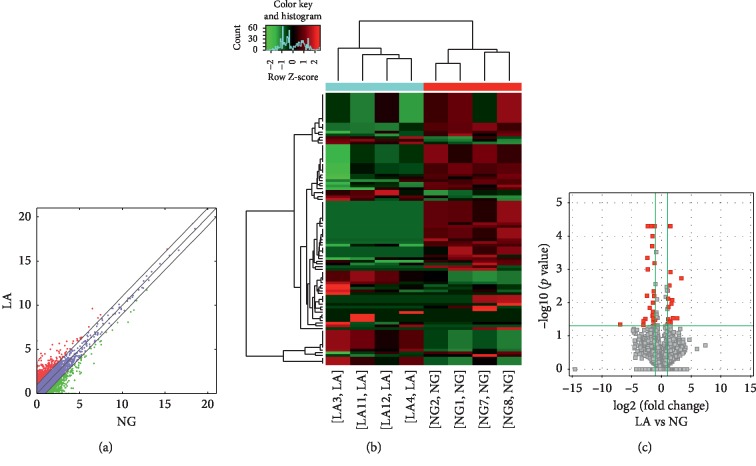
Changes in the expression profiles of lncRNAs in the LADA and nondiseased group. (a) The scatter plot reveals that a significant difference existed in the distribution of lncRNAs between the LADA group and the nondiseased group. (b) The heat map depicts the hierarchical clustering of altered lncRNAs in the LADA group compared to the nondiseased group. Red represents upregulation, while green represents downregulation. (c) The volcanic map displays the upregulated and downregulated lncRNAs in the LADA group compared to the nondiseased group. LncRNAs—long noncoding RNAs; LADA—latent autoimmune diabetes in adults.

**Figure 5:**
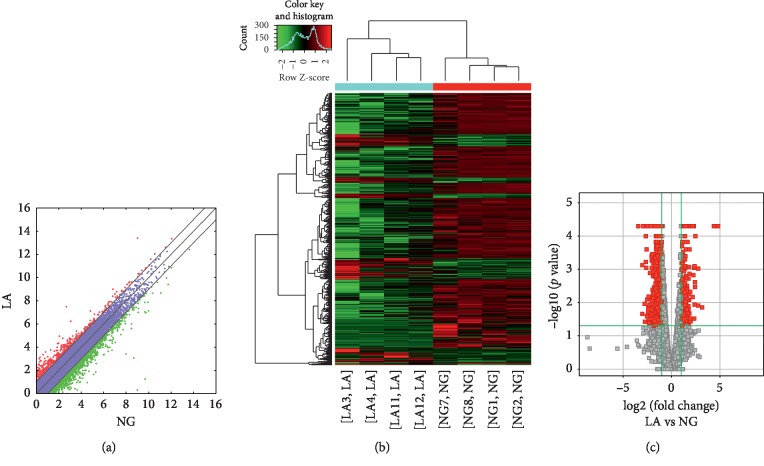
Changes in the mRNA expression profiles of the LADA and nondiseased group. (a) The scatter plot reveals that a significant difference existed in the distribution of mRNAs between the LADA group and the nondiseased group. (b) The heat map depicts the hierarchical clustering of altered mRNAs in the LADA group compared to the nondiseased group. Red represents upregulation, while green represents downregulation. (c) The volcanic map displays the upregulated and downregulated mRNAs in the LADA group compared to the nondiseased group. LADA—latent autoimmune diabetes in adults; mRNAs—messenger RNAs.

**Figure 6:**
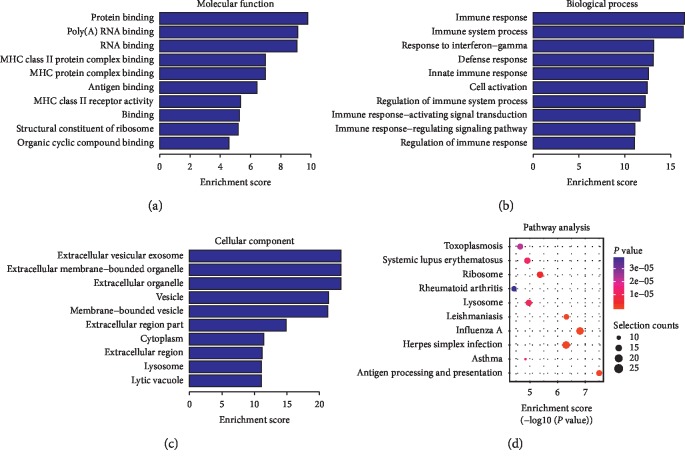
GO and KEGG signaling pathways for lncRNAs that are differentially downregulated and target mRNAs. (a) Molecular function: protein binding, poly (A)-binding RNAs. (b) Biological process: immune response and *γ*-interferon reaction. (c) Cellular component: extracellular vesicle exosomes and extracellular organelles. (d) Analysis of the KEGG signaling pathways of lncRNAs that target mRNAs: antigen processing and presentation and the signaling pathways associated with influenza A. LncRNAs—long noncoding RNAs; mRNAs—messenger RNAs; GO—gene ontology; KEGG—Kyoto Encyclopedia of Genes and Genomes.

Compared to the nondiseased group, 68,748 lncRNAs were significantly upregulated and 28,538 lncRNAs were significantly downregulated (Figures 4(a)–4(c)), while 219 mRNAs were significantly upregulated and 805 mRNAs were significantly downregulated (Figures 5(a)–5(c)) in the LADA group. Based on qRT-PCR, the expression of three lncRNAs (ENST00000602845, ENST00000424044, and ENST00000432511) were downregulated to levels representing significant fold changes. Based on GO and KEGG signaling pathways, the lncRNAs that target mRNAs are mainly involved in antigen processing and presentation, while the signaling pathways are associated with influenza A (Figure 6).

**Figure 7:**
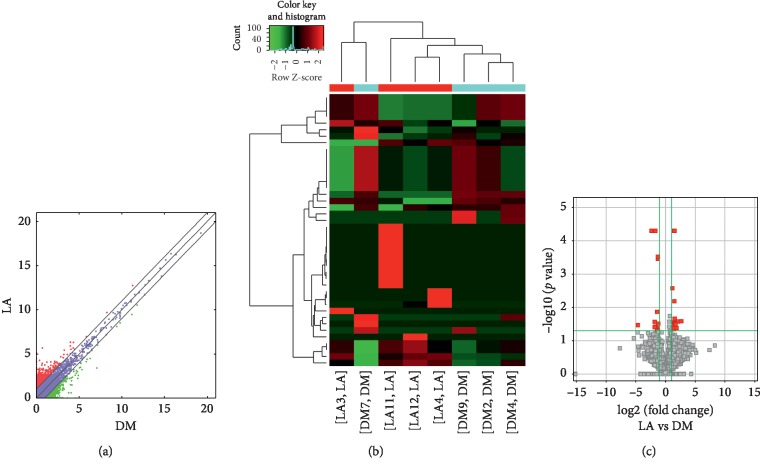
Changes in the expression profiles of lncRNAs in the LADA and T2DM groups. (a) The scatter plot reveals that a significant difference existed in the distribution of lncRNAs between the LADA group and the T2DM group. (b) The heat map depicts the hierarchical clustering of altered lncRNAs in the LADA group compared to the T2DM group. Red represents upregulation, while green represents downregulation. (c) The volcanic map displays the upregulated and downregulated lncRNAs in the LADA group compared to the T2DM group. LncRNAs—long noncoding RNAs; LADA—latent autoimmune diabetes in adults; T2DM—type 2 diabetes mellitus.

**Figure 8:**
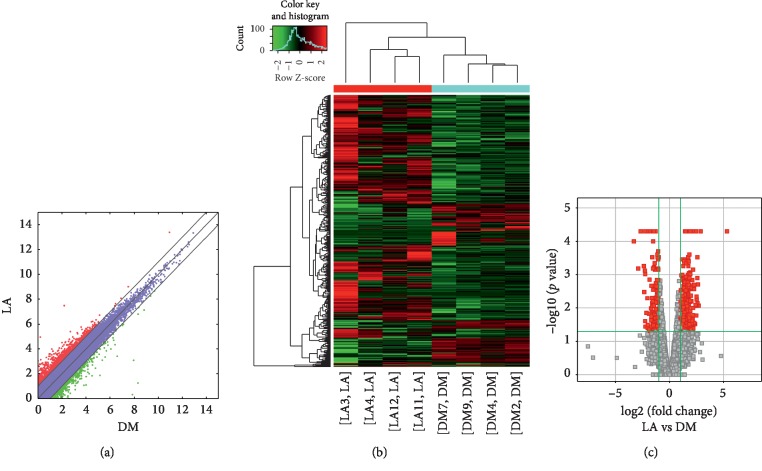
Changes in the mRNA expression profiles of the LADA and T2DM groups. (a) The scatter plot reveals that a significant difference existed in the distribution of mRNAs between the LADA group and the T2DM group. (b) The heat map depicts the hierarchical clustering of altered mRNAs in the LADA group compared to the T2DM group. Red represents upregulation, while green represents downregulation. (c) The volcanic map displays the upregulated and downregulated mRNAs in the LADA group compared to the T2DM group. LADA—latent autoimmune diabetes in adults; T2DM—type 2 diabetes mellitus; mRNAs—messenger RNAs.

**Figure 9:**
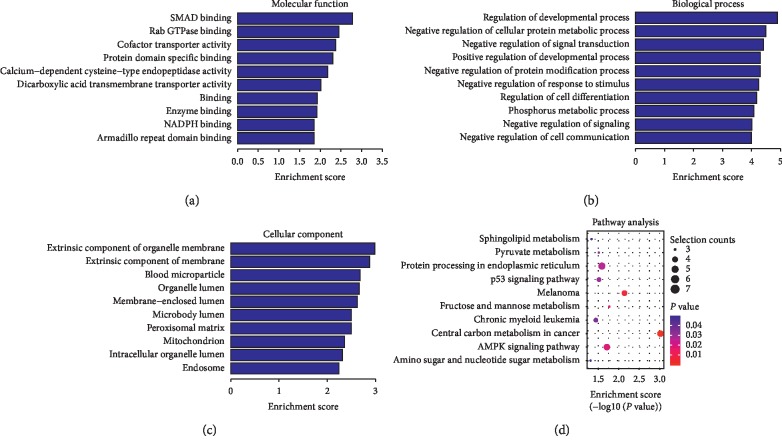
GO and KEGG signaling pathways for differentially expressed lncRNAs that target the upregulated mRNAs. (a) Molecular function: SMAD binding and Rab GTPase binding. (b) Biological process: regulation of the growth pathways and negative regulation of cellular proteins. (c) Cellular component: external components of cells and organelle membranes. (d) Analysis of the KEGG signaling pathways that target mRNAs: the intermediate carbon metabolism melanoma signaling pathway in cancer. LncRNAs—long noncoding RNAs; mRNAs—messenger RNAs; GO—gene ontology; KEGG—Kyoto Encyclopedia of Genes and Genomes.

**Figure 10:**
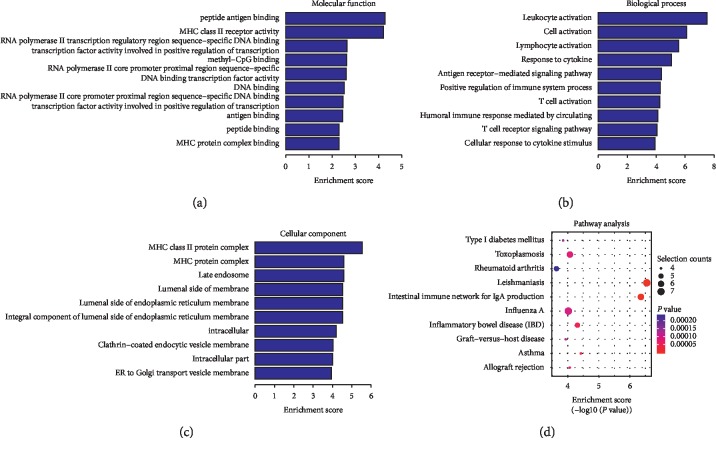
The GO and KEGG signaling pathways for the differentially expressed lncRNAs that target the downregulated mRNAs. (a) Molecular function: peptide antigen binding and MHC-II receptor activity. (b) Biological process: cell activation. (c) Cellular component: external components of cells and organelle membranes. (d) Analysis of the KEGG signaling pathways that target mRNAs: intestinal wall immune network signaling pathways for leishmaniasis and IgA products. mRNAs—messenger RNAs; GO—gene ontology; KEGG—Kyoto Encyclopedia of Genes and Genomes.

Compared to the T2DM group, 74,207 lncRNAs were significantly upregulated and 23,079 lncRNAs were significantly downregulated (Figures 7(a)–7(c)), while 349 mRNAs were significantly upregulated and 137 mRNAs were significantly downregulated (Figures 8(a)–8(c)) in the LADA group. Based on qRT-PCR, the expressions of three lncRNAs (ENST00000499762, ENST00000425189, and ENST00000436373) were downregulated to levels depicting significant fold changes. Based on GO and KEGG pathway analyses, the upregulated lncRNAs that target mRNAs are mainly involved in the intermediate carbon metabolism melanoma signaling pathway in cancer (Figure 9), while the downregulated lncRNAs that target mRNAs are mainly involved in intestinal wall immune network signaling pathways for leishmaniasis and IgA products (Figure 10).

**Figure 11:**
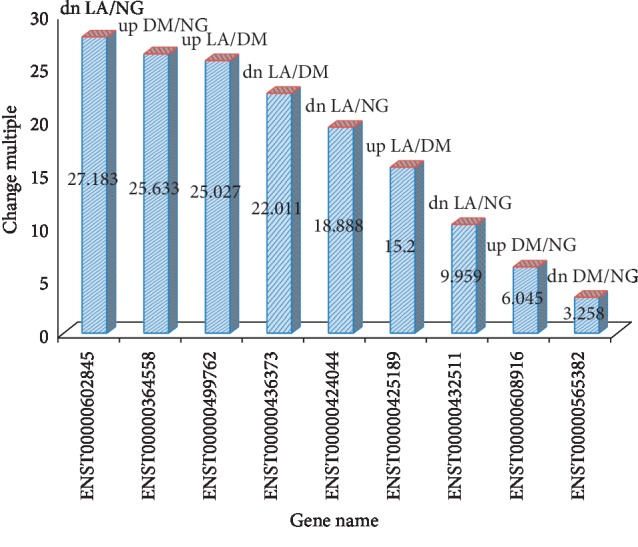
The result diagram of qRT-PCR.

For validation purposes, we selected four upregulated and five downregulated lncRNAs to identify the differentially expressed lncRNAs by qRT-PCR (Table 2). All primer sequences are listed in Table 2. The qRT-PCR results were consistent with our sequencing results for the upregulated and downregulated lncRNAs (Figure 11).

## 4. Discussion

### 4.1. Latest Development of lncRNAs

Owing to the development of the high-throughput sequencing technique, there has been an increasing evidence that only less than 2% of the entire human genome belongs to protein-coding genes and most of the genome contains ncRNAs [2]. NcRNAs may regulate the development of complex organisms, while lncRNAs, which are a type of newly discovered and ubiquitous ncRNAs, have diverse functions and a length exceeding over 200 nucleotides. LncRNAs are transcribed by RNA polymerase II. In fact, most are enriched in the cytoplasm, while some are partially enriched in the nucleus or concurrently enriched in the nucleus and cytoplasm [8]. Initially, lncRNAs were viewed as “junk genes” that lack coding capacity [9]; however, they were discovered to be important in many human diseases such as diabetes, cancer, neurodegenerative diseases, cardiovascular diseases, and autoimmune diseases, such as T1DM [10, 11]. LncRNAs can interact with DNA, proteins, mRNAs, miRNAs, siRNAs, and other antisense RNAs via chromatin modification [8, 12], thereby acting as a bait or promoter of transcription factors [4], DNA methylation, histone modification, and genomic imprinting [13] and promoting or inhibiting translation; these interactions are involved in gene expression and regulation [14].

### 4.2. The Relationship between lncRNAs and DM

Numerous studies have found changes in some lncRNAs that are associated with diabetes [15]. Following decades of mouse studies and genome-wide association studies, Scott et al. identified several genetic drivers of *β* cell dysfunction in monogenic syndrome, 113 susceptible sites of T2DM, and 60 loci associated with the increased risk of T1DM [16]. Most T1D and T2D susceptible loci are located outside the coding region of the gene, suggesting that noncoding genes have a crucial role in the development of DM [17]. As a result, we extracted blood samples from four cases of T2DM, four cases of LADA, and four nondiseased adults for high-throughput sequencing. Systematic processing and analysis of the experimental data revealed that a significant difference existed in the variation of genes among the T2DM, LADA, and nondiseased groups. In addition, a statistical significance was found for the differentially expressed lncRNAs, mRNAs, and target genes. Nine differentially expressed lncRNAs, four upregulated and five downregulated lncRNAs, were selected for qRT-PCR. The qRT-PCR results were found to align with the sequencing results for the upregulated and downregulated lncRNAs. Furthermore, a total of seven significantly coexpressed gene pairs, namely, BTG2, A2M, HECTD4, MBTPS1, DBH, FLVCR1, and NCBP2, were obtained, and two lncRNAs (ENST00000608916 and ENST00000436373) were found to have no coexpression relationship as a pair for the time being.

### 4.3. Analysis of the Experimental Results

Based on the above-mentioned theory, blood samples from four cases of T2DM patients, four cases of LADA patients, and four nondiseased adults were retrieved for high-throughput sequencing. Through systematic processing and an analysis of the experimental data, we found that a significant difference existed in the variation of genes among the T2DM, LADA, and nondiseased groups. In addition, a statistical significance was found for the differentially expressed lncRNAs, mRNAs, and target genes. Most lncRNAs act as cis-regulatory factors owing to the significant association between their expression and their adjacent protein-coding genes [18]. Therefore, to derive the role of differentially expressed lncRNAs in the pathogenesis of T2DM and LADA, we screened related genes according to the regulatory relationship between lncRNAs and mRNAs.

### 4.4. Data Comparison between the T2DM and Nondiseased Groups

The high-throughput sequencing results revealed that HECTD4 (C12orf51|POTAGE), which encodes E3 ubiquitin-protein ligase 4, was the coexpressed gene for the significantly upregulated lncRNA, ENST00000364558. HECTD4 was also identified to control substrate specificity in the form of E3s. Ubiquitination is a posttranslational modification involved in biological processes such as cell cycle, apoptosis, transcription, protein transport, signal transmission, DNA replication and repair, and angiogenesis [19]. Previously, Seongwon et al. verified that the two single nucleotide polymorphisms in HECTD4 were significantly associated with the decrease in bust-to-hip ratio, which may cause insulin resistance through inflammation, ultimately resulting in T2DM [20]. GO analysis revealed that cell membrane components play a role in ubiquitin-protein transferase and ligase activity in biological processes, such as protein ubiquitination and glucose metabolism. As a result, the target gene of ENST00000364558 was predicted to be HECTD4, which may be involved in T2DM development via the phagosome signaling pathway. The expression level of ENST00000565382 was significantly downregulated, and MBTPS1 (PCSK8|S1P|SKI-1) was identified as its coexpressed gene via high-throughput sequencing. The gene encoding the proprotein-transferase family of subtilisin, which is localized to the cis/medial Golgi apparatus, regulates the homeostasis of cholesterol or lipid at alkaline residues via substrate lysing and plays an important role in regulating the formation of somatic cell division [21]. Following a review of the literature, the gene encoding S1P was speculated to affect the function of lysosomes and result in the development of DM. GO analysis revealed that vesicles are involved in the immune response during immunoglobulin binding. MBTPS1 was predicted to be the target gene of ENST00000565382 and may be involved in the occurrence of T2DM through the lysosome and phagocytic signaling pathways. The expression level of ENST00000608916 was upregulated, and the target gene was predicted to be HCCS per the proximity correlation, thereby encoding mitochondrial holocytochrome c synthase, with cytochrome c as the active product. During persistent and severe endoplasmic reticulum stress and oxidative stress, the release of reactive oxygen species is increased, and a high level of cytochrome c release from the mitochondria activates caspases, ultimately initiating apoptosis in cells [22]. GO analysis revealed that the hemoglobin complex triggers erythrocyte development and transfers gas in biological processes such as oxygen transportation and oxygen binding. The mRNA transcribed from this target gene may participate in the occurrence of T2DM through the phagosome signaling pathway.

### 4.5. Data Comparison between the LADA and Nondiseased Groups

For the downregulated intergenic lncRNA, ENST00000432511, the results of high-throughput sequencing revealed BTG2 (PC3|TIS21) as its coexpressed gene. BTG2 encodes antiproliferative factor 2, which is a transcriptional synergistic regulatory factor involved in the regulation of cell differentiation, growth, and survival [23]. The expression of PDX-1 in *β* cells positively regulates GLP-1 to stimulate pancreatic *β* cell gene expression and secretion. Thus, the GLP-1-BTG 2-PDX-1 network may provide a new molecular mechanism for controlling insulin gene expression and may become an important factor that regulates insulin secretion [24]. Shi et al. found that BTG2, a controlling gene in cell cycle, cell proliferation, and cell death, is involved in the regulation of G1/S transition in the cell cycle [25]. Through GO analysis, extracellular bodies were demonstrated to participate in the proximal sequence specific binding and inhibition of the transcriptional activity of the RNA polymerase II core promoter in the RNA degradation process. BTG2 is thus speculated to be the target gene of lncRNA ENST00000432511 and may be involved in the pathogenesis of diabetes via the RNA degradation signal pathways. Based on the high-throughput sequencing data, NCBP2 (CBC2|CBP20|NIP1|PIG55), which encodes the nuclear cap binding protein subunit 2, was identified as the coexpressed gene of the significantly downregulated lncRNA ENST00000602845. In addition, GO analysis revealed that the cap structure of the mRNA plays a monitoring role in mRNA polyadenylation and shearing [26]. NCBP2 is speculated to be the target gene, and it participates in the occurrence of DM through the mRNA monitoring and splicing signaling pathways.

### 4.6. Data Comparison among the T2DM/Nondiseased Group, LADA/Nondiseased Group, and LADA/T2DM Group

As lncRNA ENST00000499762 was upregulated in LADA and T2DM, these diseases are believed to share a common pathogenic gene. In fact, high-throughput sequencing indicated that the coexpressed gene, A2M (A2MD|CPAMD5|FWP007|S863-7) which encodes the *α*-2-macroglobulin that acts as a carrier of proinflammatory cytokines such as interleukin-1*β*, interleukin-6, and tumor necrosis factor-*α* [27], causes diabetic retinopathy [28]. GO analysis revealed that exosomes are involved in calcium-dependent protein and protease binding during blood coagulation. A2M is presumably the target gene and may participate in the occurrence of DM through the complement cascade signaling pathway. lncRNA ENST00000424044 is downregulated in both LADA and T2DM, which may share a common pathogenic mechanism. Through high-throughput sequencing, FLVCR1 (AXPC1|FLVCR|MFSD7B|PCA|PCARP), which encodes cat leukemia virus C subpopulation cell receptor 1 (FLVCR1), a heme transporter involved in different biological processes such as cell proliferation, cell death, apoptosis, oxidative stress, cell differentiation, and metabolism, was identified as the coexpressed gene. Increasing evidence indicates that the absence of FLVCR1 stimulates FLVCR1a to export excess free heme, which leads to heme accumulation in erythroid progenitor cells of the liver and intestinal tissues, and triggers the corresponding increase in heme oxygenase 1. These processes promote the formation of reactive oxygen species and iron-induced oxidative stress and lead to *β* cell apoptosis, liver dysfunction, and insulin resistance, ultimately promoting the progression of T2DM [29, 30]. GO analysis revealed that the cell membrane components participate in the regulation of heme transport activity during heme transport. FLVCR1 is speculated to be the target gene of lncRNA ENST00000424044. In fact, it participates in the pathogenesis of DM via immunoregulation. In this study, lncRNA ENST00000425189 was upregulated in the LADA group relative to the T2DM group and in the LADA group relative to the nondiseased group but downregulated in the T2DM group relative to the nondiseased group. Therefore, ENST00000425189 may be involved in the pathogenesis of LADA via upregulation and T2DM via downregulation. High-throughput sequencing revealed that DBH (DBM), which encodes dopamine *β*-hydroxylase, a copper type II oxidoreductase, for the catalysis of dopamine's conversion to norepinephrine [31, 32], was the coexpressed gene. Ailanen et al. found that the functional polymorphism of the human neuropeptide Y gene (Rs 16139) promotes the secretion of neuropeptide Y; inhibits insulin release through the pancreatic Y1 receptor; suppresses sympathetic nerve activity, energy expenditure, and heat production of brown adipose tissue; and increases the production of white fat tissue and insulin resistance, ultimately triggering hyperinsulinemia and impairing glucose tolerance. Once pancreatic insulin secretion is interrupted, the onset of T2DM is expected [33]. Based on GO analysis, the secretory vesicle membrane participated in copper ion binding during synaptic transmission, and DBH is speculated to be the target gene of lncRNA ENST00000425189, which may be involved in DM through the tyrosine metabolic pathway. As lncRNA ENST00000436373 was downregulated in the LADA group compared to the nondiseased group or in the LADA group compared to the T2DM group but upregulated in the T2DM group compared to the nondiseased group, its downregulation and upregulation may be associated with the pathogenesis of LADA and T2DM, respectively. However, its target gene has not been identified but is speculated to be an mRNA that displays a significant differential expression. Based on GO/KEGG pathway analysis, the external components of cells and organelle membranes may participate in the development of diabetes through the immune network signaling pathways.

In conclusion, further investigations are warranted to derive the difference in gene expression among T2DM, LADA, and nondiseased groups. However, a statistical significance in the lncRNA target genes can provide a gene profile for the diagnosis of DM, ultimately achieving an accurate treatment for this disorder. To date, most studies on lncRNAs have focused on large-scale discovery using genome-wide methods. However, the mechanism and functional analysis of lncRNAs remain unknown. In the near future, deriving lncRNA-based transcriptional signals may provide a new insight into the diagnosis, classification, or personalized treatment of LADA in immune-mediated and inflammatory diseases. Gomes et al. reported that the pathogenesis of obesity-related T2DM is mainly related to the differentiation of inflammatory immune cells in the adipose tissue, including macrophages, T-cells, B cells, and neutrophils, further leading to insulin resistance. Such findings may contribute to the treatment of inflammatory diabetic complications based on lncRNA. Current studies have indicated that lncRNAs are important regulators of DM and are thus likely to serve as important diagnostic and therapeutic targets for this disorder. Therefore, an in-depth study on lncRNAs will be rather challenging but will reveal their potential and role in the area of therapeutics.

### 4.7. Research Limitations

1. The experiment covers two diseases, but the number of patients and the number of samples collected were small and thereby limited the reference significance.
2. There are three possible reasons for the failure to discover the coexpressed genes. First, lncRNA is a competitive endogenous RNA that remotely regulates genes at the posttranscriptional level. As a result, its target gene is unlikely to be derived from adjacent protein-encoding genes. Second, in recent studies, some lncRNAs were found to directly encode peptides by acting on their adjacent genes, ultimately regulating biological processes. Third, the target gene of the lncRNAs was not located at the predicted site in the present study; thus, gene prediction must be comprehensively conducted by expanding the search range of the target gene loci.

## Figures and Tables

**Table 1:** Primers for lncRNAs.

Genes	Type of primers	Sequences (5′-3′)
ENST00000364558	Forward	ATCTGTCACCCCATTGATCG
	Reverse	AGACCGGTCCTCCTCTATCG
ENST00000436373	Forward	GAGGCCTGGTGTGGAGTTAG
	Reverse	CTGGTTTGATTCACGCACAC
ENST0000042404	Forward	TGCTGATGTGCCCACTAAAG
	Reverse	CCATCATAGCCCGTCTCAGT
ENST00000432511	Forward	AGACGGTTCCCCTTTCTGAT
	Reverse	TCTACCGAGTGCCTGTGATG
ENST00000602845	Forward	TTTCCTCTGCAAGTGGGACT
	Reverse	CATGCCTATCAACCAGCTCA
ENST00000608916	Forward	TGGTTATGCCTGGAGACCTT
	Reverse	ACCCAGCTTCAAACATCTGG
ENST00000425189	Forward	TTTGCCAGCATTGTTCTCTG
	Reverse	CGGACTCAGTTCCCTTTTGA
ENST00000565382	Forward	TGTCATGGACTCGTGGAGAG
	Reverse	GCCATCTTAACGAGCTACCG
ENST00000499762	Forward	GAACAGGACTCCAGCAAAGC
	Reverse	GGGCACTATGCTACCCAGAA
ACTB	Forward	GTGGATCAGCAAGCAGGAGT
	Reverse	AAAGCCATGCCAATCTCATC

**Table 2:** The lncRNAs that are significantly upregulated and downregulated.

Transcript_id	Regulation	log2 (fold change)	*p*_Value	Chr	Strand	Start	End
ENST00000364558	Up	3.35535	0.01445	12	+	112597991	112819896
ENST00000608916	Up	2.51016	0.03675	X	−	11129405	11141204
ENST00000565382	Down	-inf	0.00005	16	+	84135339	84150440
ENST00000425189	Up	2.54798	0.00305	9	−	136501484	136524466
ENST00000424044	Down	-inf	0.00635	1	−	213031596	213072705
ENST00000432511	Down	-inf	0.0303	1	−	203274663	203278729
ENST00000499762	Up	1.26756	0.00265	1	+	9220259	9244008
ENST00000602845	Down	−2.62767	0.02675	3	+	196662272	196669464
ENST00000436373	Down	-inf	0.00005	21	−		

## Data Availability

The data used to support the findings of this study are available from the corresponding author upon request.

## Abbreviation

LncRNAs: Long noncoding RNAs
mRNAs: Messenger RNAs
DM: Diabetes mellitus
T2DM: Type 2 diabetes mellitus
LADA: Latent autoimmune diabetes in adults
FPKM: Fragments per kilobase of exon model per million reads mapped
T1DM: Type 1 diabetes mellitus
GO: Gene ontology
KEGG: Kyoto Encyclopedia of Genes and Genomes
ncRNAs: Noncoding RNAs
SNPs: Single-nucleotide polymorphisms.
